# What is the level of nutrition care provided to older adults attending emergency departments? A scoping review protocol.

**DOI:** 10.12688/hrbopenres.13485.1

**Published:** 2022-01-18

**Authors:** Anne Griffin, Lorna Ryan, Mairéad Conneely, Sheila Bowers, Liz Dore, Rose Galvin

**Affiliations:** 1 School of Allied Health, Faculty of Education and Health Sciences, University of Limerick, Limerick, V94 T9PX, Ireland; 2 Ageing Research Centre, Health Research Institute, University of Limerick, Limerick, V94 T9PX, Ireland; 3 Department of Clinical Nutrition & Dietetics, University of Limerick Hospital Group, Dooradoyle, Limerick, V94 F858, Ireland; 4 Glucksman Library, University of Limerick, Limerick, V94 T9PX, Ireland

**Keywords:** Emergency medicine; integrated care pathways; malnutrition; nutrition and dietetics; older adults; nutrition care process

## Abstract

**Introduction:** Nutrition status among older adults is an important factor in health and clinical outcomes but malnutrition goes unrecognised in routine health care.  Older adults often present to emergency departments (ED) and are subsequently discharged without hospital admission.  Discharge is a transitionary time of care when nutritional vulnerability could be mitigated with the instigation of targeted nutrition care pathways. This protocol outlines a scoping review to identify the level of nutrition care provided to older adults attending emergency departments.

**Methods:** This scoping review will be conducted using the framework proposed by the Joanna Briggs Institute. The Preferred Reporting Items for Systematic Reviews and Meta-analysis extension for scoping reviews (PRISMA-ScR) will be used to guide the reporting. Two researchers will search electronic databases (Medline, CINAHL Complete, EMBASE, Cochrane Library and Scopus), grey literature sources (DART-Europe E-theses portal, Open Grey, and Trip Medical database) and website searches (Google, Google Scholar, Pubmed, NICE and LENUS) to identify appropriate data for inclusion within the last 10 years. Key information will be categorised and classified to generate a table charting the level of nutrition and dietetic care initiated for older adults in the ED according to the Nutrition Care Process Model. A narrative synthesis will be conducted.

**Conclusions: **This scoping review will be used to inform a foundational concept of nutrition care in an ED setting and allow the future examination of nutrition care pathways, practice, policy, and research within models of integrated care for older persons.

## Introduction

The process of ageing has an impact on the nutritional status of an individual. This can occur due to factors such as the physiological (e.g. taste changes, poor dentition, loss of appetite, mobility and functional limitations), psychosocial (e.g. life course, food ideals and preferences, grief and bereavement), and personal resources (e.g. transport, disposable income, social supports) that influence food choice and intake ( Host *et al.*, 2016; Stanga, 2009) among older adults. In addition to ageing, older adults are more likely to be living with one or more non-communicable chronic diseases ( Shlisky *et al.*, 2017). These diseases can independently influence or be influenced by nutritional status ( Shlisky *et al.*, 2017; Slawson *et al.*, 2013; Stanga, 2009; Tittikpina *et al.*, 2019).

A nutritional vulnerability can be described as a reduced physical reserve of energy and protein that limits an individual’s ability to recover sufficiently from an acute health threat ( Starr *et al.*, 2015). The term malnutrition describes a state of under or over nutrition of energy, protein and/or micronutrients ( Cederholm *et al.*, 2017). This state may be caused by reduced food and nutrient intake and/or assimilation of nutrients from the digestive system and/or inflammatory mechanisms associated with acute and chronic disease ( Cederholm *et al.*, 2019). 

A failure to identify malnutrition in the continuum of older adult care, particularly transitions from hospital to community settings, has been described to increase the risk of nutrition vulnerability ( Starr *et al.*, 2015). The Nutrition Care Process Model (NCPM) has been adapted by national dietetic associations and implemented within healthcare organisations to provide a standardised process, terminology and dietetic outcome frameworks toward person-centered nutrition care and outcomes management ( Swan *et al.*, 2017; Swan *et al.*, 2019). Screening is described as the first step to identify “at risk” of malnutrition status with the use of a validated malnutrition screening tool. This process serves to identify those who require targeted assessment and nutrition interventions ( Cederholm *et al.*, 2019; Swan *et al.*, 2017; Swan *et al.*, 2019)

Malnutrition is linked with aging-related disease and is a significant cause for hospitalisation among older adults ( Hong *et al.*, 2019; Tittikpina *et al.*, 2019). In particular, malnutrition plays a role in the development and progression of frailty and sarcopenia ( Cruz-Jentoft *et al.*, 2017). Research demonstrates that older adults are frequent users of emergency departments (ED), accounting for up to one quarter of all ED attendees ( Morley *et al.*, 2018; Roe *et al.*, 2018; van Tiel *et al.*, 2015). We have previously reported finding over a third of non-acute older adults admitted and subsequently discharged from ED to be at risk of malnutrition or malnourished when screened with the Mini Nutritional Assessment – Short Form (MNS-SF) tool ( Griffin *et al.*, 2020). However, nutrition screening is not routinely performed in ED even when mandated by clinical guidelines due to perceived demands on nursing time to perform the screening, different priorities relating to patient flow, and individual barriers relating to practitioners’ competency ( Dent *et al.*, 2019; Kirk & Nilsen, 2016; Vivanti *et al.*, 2015). Therefore, there are missed opportunities to initiate integrated care pathways to ameliorate nutrition vulnerability ( Starr *et al.*, 2015; Umegaki *et al.*, 2017; Vivanti *et al.*, 2015). 

The purpose of this proposed scoping review is to identify the extent of nutrition care provided to older adults attending and subsequently discharged from ED. This information will be used to inform a foundational concept of nutrition care according to the NCPM in an ED setting and allow the future examination of nutrition care pathways, practice, policy, and research within models of integrated care for older adults. The research question for this scoping review is:

What is the level of nutrition care provided to older adults attending emergency departments?

An initial search of MEDLINE, the Cochrane Database of Systematic Reviews and *JBI Evidence Synthesis* was conducted and there were no current or underway systematic reviews or scoping reviews and few empirical research articles on the topic identified. A preliminary Google search found articles reported in healthcare professional journals, reports, and websites. Therefore, a scoping review has been chosen to explore the breadth of grey and published literature to provide a holistic synthesis of evidence and identify research gaps and focus for future studies.

## Methods

This scoping review will be conducted in accordance with the Joanna Briggs Institute (JBI) methodology for scoping reviews ( Peters *et al.*, 2020). The preferred reporting items for systematic reviews and meta-analysis extension for scoping reviews (PRISMA-ScR) will be used to guide the report ( Tricco *et al.*, 2018). As this is a scoping review it will be designed to explore the breadth and depth of the literature represented as a tabular map that summarises the evidence and activity related to nutrition care in the ED among older adults ( Cooper, 2016; Tricco *et al.*, 2016).

The research question was identified and the stated objectives refined from the preliminary searches and consultation with academic colleagues (RG, MCC) engaged in the exploration of the roles of dedicated health and social care professionals in the care of older adults in the ED ( Cassarino *et al.*, 2019; Conneely *et al.*, 2021; O'Shaughnessy *et al.*, 2019) and registered dietitians (SB, LR) engaged in the service provision of nutrition care for older adults in acute and frailty intervention teams. The following objectives were developed to guide the scoping review:

1. To explore current screening practices and tools used to identify malnutrition risk among older adults who present to ED and up to 72-hours upon discharge to home.
2. To map the current levels of nutrition and dietetic care provided to older adults who present to ED and up to 72-hours upon discharge to home.
3. To describe the pathways of nutrition care initiated on identification of (risk of) malnutrition for older adults discharged from the ED.

## Eligibility criteria

The mnemonic PCC (population, concept, and context) was adopted to guide the development of the inclusion criteria, search terms and strategy for scoping reviews ( Peters *et al.*, 2020).

### Population

Older male and female adults (those with a mean age ≥65 years) who present to the ED and are subsequently discharged from emergency departments up to 72-hours post discharge to include early discharge planning phase.

### Concept

The phenomena of interest are nutrition focused screening and subsequent level of nutrition care provided to manage malnutrition initiated from an ED setting. The nutrition screening may be conducted independently or as part of a comprehensive geriatric assessment. The nutrition screening can be carried out by any member of the ED multidisciplinary team (MDT). Subsequent nutrition care must be initiated at presentation to the ED, during the stay in the ED index visit or up to 72 hours post ED discharge. We are not primarily interested in patient outcomes for this review but will chart these as a secondary finding.

### Context

The search will be limited to publications describing nutrition screening, assessment, diagnosis, nutrition interventions, monitoring and/or evaluation in ED settings within the last 10 years to ensure currency to present day. Studies will be confined to those from developed countries. Studies that evaluate any nutrition intervention will be included. Medication studies will be excluded.

***Inclusion criteria***

- Literature published in last 10 years (2011–2021).
- Grey literature including studies, reports and published articles that focus on nutrition assessment tools to measure nutrition status and intervention among older adults (65+ years) in the ED.
- Articles published in any language.
- Studies that report on nutrition screening and subsequent nutrition care by any MDT member or a qualified registered dietitian/clinical nutritionist.
- Review articles including systematic reviews, scoping reviews, and rapid reviews; quantitative studies (observational and experimental), qualitative and mixed method studies, and clinical care guidelines.
- Grey literature defined as “that which is produced on all levels of government, academics, business and industry in print and electronic formats, but which is not controlled by commercial publishers” ( Frederiksen, 2008).

***Exclusion criteria***:

- Articles published before January 2011.
- Articles focusing on ages less than a mean of 65 years admitted in the ED.

## Search strategy

The search strategy for this scoping review was developed in collaboration with a specialist librarian (LD) who carried out an initial search ( Table 1). The search strategy will aim to locate both published and unpublished studies and will be iterative through three steps:

**Table 1.** Search terms of inclusion criteria according to PCC (population, concept, and context) mnemonic ( Peters *et al.*, 2020).

Database search terms
Participants: older adults or elderly or geriatric or geriatrics or aging or senior or seniors or older people or aged 65 or 65+ or retired Concept: nutrition screen* or malnutrition or nutritional status or nutrition assess* Context: emergency department or emergency room or accident and emergency or accident & emergency or a&e or a & e or Casualty department or triage in the emergency department or triage or triage system or trauma cent* or emergency services or A & E or A&E

1. An initial limited search of CINAHL was undertaken to identify articles on the topic (see *Extended data* ( Griffin, 2021)). The text words contained in the titles and abstracts of relevant articles, and the index terms used to describe the articles will be used to develop a full search strategy with the input of a specialist health sciences librarian (LD).
2. The search strategy, including all identified keywords and index terms, will be adapted for each included database and/or information source. Two researchers (AG, LD) will search electronic databases (Medline ( Ovid), Pubmed, CINAHL Complete, EMBASE, Cochrane Library and Scopus), grey literature sources ( DART-Europe E-theses portal, Open Grey, and Trip Medical database) and website searches (Google, Google Scholar, NICE and LENUS) for relevant professional and organisational developed policy, practice and guidelines.
3. The reference list of all included sources of evidence will be screened for additional studies. As the search for scoping reviews is an iterative process as researchers become more familiar with the evidence and identify additional keywords, sources, and search terms the entire search strategy and results will be reported in detail with the published review.

### Evidence selection

Following the search, all identified citations will be collated and uploaded into EndNote X8 and duplicates removed. The screening process will be carried out using Rayyan open access screening software ( Ouzzani *et al.*, 2016). Study selection will begin with screening of titles and abstracts by two reviewers (AG and RG), independently, using the pre-specified inclusion and exclusion criteria. The screening process will be pilot tested on a random sample of 25 titles and abstracts. Subsequently, the full text of selected citations will be assessed in detail against the inclusion criteria by two independent reviewers (AG and LR). Reasons for exclusion of sources of evidence at full text that do not meet the inclusion criteria will be recorded and reported in the scoping review. Any disagreements that arise between the reviewers at each stage of the selection process will be resolved through discussion, or with an additional reviewer (RG or MC). The results of the search and the study inclusion process will be reported in full in the final scoping review and presented in a Preferred Reporting Items for Systematic Reviews and Meta-analyses extension for scoping review (PRISMA-ScR) flow diagram ( Tricco *et al.*, 2018).

### Data extraction

An adapted data extraction tool from the template provided by the JBI methodology guidance for scoping reviews ( Peters *et al.*, 2020a) will be used for collation. The data extracted will include specific details about the participants, concept, context, article type, country, and study methods as relevant. A draft extraction form is provided ( Table 2). The draft data extraction form will be modified and revised as necessary during the process of extracting data from each included evidence source. As part of this process one reviewer will independently chart the data from the retrieved articles (AG). The second reviewer (MC) will check a sample of 20% of the charted data. Any disagreements that arise between the reviewers will be resolved through discussion, or with additional reviewers (RG). If appropriate, authors of papers will be contacted to request missing or additional data, where required. Subsequent modifications will be detailed in the scoping review. Data charting will be conducted using Microsoft Excel Version 2111.

**Table 2.** Adapted data charting form from Joanna Briggs Institute (JBI) methodology for scoping reviews ( Peters *et al.*, 2020a).

**Scoping review details**
Scoping review title
Scoping review objectives
Scoping review questions
**Inclusion/Exclusion criteria**
Participants
Concept
Context
Type of evidence source
**Evidence Source Details and Characteristics**
Citation details (author(s), date, title, journal, volume, issue)
Country
Context (admitted to ED or discharged home from ED (up to 72 hours), length of ED stay, etc.)
Participants (details of age/sex and number)
**Details/Results extracted from source of evidence** (in relation to the concept of the scoping review)
Nutrition screening (completed (Y/N), tool used, independent/part of CGA, role of healthcare professional completing screening)
Nutrition assessment (completed (Y/N), detail of assessment (assessment tool/clinical exam/etc.), independent/part of CGA, MDT member completing assessment)
Nutrition diagnosis (description, use of standardised language (Y/N), documentation in health care record)
Nutrition intervention (description including prescription of oral nutritional supplements, etc.)
Nutrition monitoring and evaluation (frequency, responsibility, etc.)
Referral to/from care pathways supporting transitionary nutrition care for older adults

*Abbreviations Legend. ED, emergency department; Y/N, yes/no; CGA, comprehensive geriatric assessment; MDT, multidisciplinary team.*

Key findings relevant to the review question and objectives will describe data according to the steps of the NCPM and subsequent referral for nutrition care follow-up ( Table 2).

A critical appraisal of methodological quality or risk of bias of included studies is not applicable as the purpose of this scoping review is to describe current practices in malnutrition screening among older adults attending and subsequently discharged from an ED and to map the levels of nutrition care from data that spans the evidence hierarchy ( Peters *et al.*, 2020).

### Data analysis and presentation

Data analysis will be conducted using SPSS Version 24. The data will be presented in tabular form and will include basic descriptive analysis (i.e. frequency counts of concepts, population, etc.). Qualitative data gathered will also be presented as a descriptive narrative and it is beyond the remit of a scoping review to perform a thematic analysis ( Peters *et al.*, 2020). However, basic coding of data will be performed to identify and map the steps (Screening, Assessment, Diagnosis, Monitoring and Evaluation) of the Nutrition Care Process ( Harfield *et al.*, 2018; Swan *et al.*, 2017; Swan *et al.*, 2019). A narrative summary will accompany the tabulated results and will describe how the results relate to the research question and objectives.

## Dissemination

We intend to disseminate the results through publication in a peer-reviewed journal and conference presentations. We will present our findings to registered dietitians engaged in the service provision of nutrition care for older adults in acute and frailty intervention teams. We will also present our findings to a stakeholder panel of older adults for health services research to gain their insight and input to follow on research in this area including but not limited to prospective study to explore the development of integrated nutrition care pathways from the ED.

## Study status

This protocol has been finalised by the research team and was registered prospectively with the Open Science Framework on 07/01/2022 (see *Extended data* ( Griffin, 2021). At the time of publication, initial searches of databases have commenced.

## Discussion

The purpose of this proposed scoping review is to identify and map the level of nutrition care provided to non-urgent older adults attending emergency departments. ED discharge is a transitionary time of care when nutritional vulnerability could be mitigated with the instigation of targeted nutrition care pathways. This information will be used to inform a foundational concept of nutrition care according to the Nutrition Care Process in an ED setting and allow the future examination of nutrition care pathways, practice, policy, and research within models of integrated care for older persons.

## Data availability

### Underlying data

No data are associated with this article.

### Extended data

Open Science Framework: https://doi.org/10.17605/OSF.IO/CXARF ( Griffin, 2021).

This project contains the following extended data:

- Search Strategy Scoping review Nutrition Care in ED.pdf
- PRISMA-ScR-Fillable-Checklist_10Sept2019 ED Nutrition Care Protocol.docx
- ED Plus Nutrition Care Scoping Review protocol - HRB Open.pdf

Data are available under the terms of the Creative Commons Attribution 4.0 International license (CC-BY 4.0).

## References

[ref-1] Cassarino M Robinson K Quinn R : Impact of early assessment and intervention by teams involving health and social care professionals in the emergency department: A systematic review. *PLoS One.* 2019;14(7):e0220709. 10.1371/journal.pone.0220709 31365575 PMC6668840

[ref-2] Cederholm T Barazzoni R Austin P : ESPEN guidelines on definitions and terminology of clinical nutrition. *Clin Nutr.* 2017;36(1):49–64. 10.1016/j.clnu.2016.09.004 27642056

[ref-3] Cederholm T Jensen GL Correia MITD : GLIM criteria for the diagnosis of malnutrition - A consensus report from the global clinical nutrition community. *Clin Nutr.* 2019;38(1):1–9. 10.1016/j.clnu.2018.08.002 30181091

[ref-4] Conneely M Robinson K Leahy S : Effectiveness of interventions to reduce adverse outcomes among older adults following emergency department discharge: Protocol for an overview of systematic reviews [version 2; peer review: 2 approved]. *HRB Open Res.* 2021;3:27. 10.12688/hrbopenres.13027.2 33969262 PMC8078215

[ref-5] Cooper ID : What is a "mapping study?". *J Med Libr Assoc.* 2016;104(1):76–78. 10.3163/1536-5050.104.1.013 26807058 PMC4722648

[ref-6] Cruz-Jentoft AJ Kiesswetter E Drey M : Nutrition, frailty, and sarcopenia. *Aging Clin Exp Res.* 2017;29(1):43–48. 10.1007/s40520-016-0709-0 28155181

[ref-7] DentE HoogendijkEO VisvanathanR : Malnutrition screening and assessment in hospitalised older people: a review. *J Nutr Health Aging.* 2019;23(5):431–441. 10.1007/s12603-019-1176-z 31021360

[ref-8] Frederiksen L : Grey Literature Report. *Reference Reviews.* 2008. Reference Source

[ref-9] Griffin A : Nutrition care in emergency departments.2021. 10.17605/OSF.IO/FCQSY

[ref-10] Griffin A O’Neill A O’Connor M : The prevalence of malnutrition and impact on patient outcomes among older adults presenting at an Irish emergency department: a secondary analysis of the OPTI-MEND trial. *BMC Geriatr.* 2020;20(1):455. 10.1186/s12877-020-01852-w 33160319 PMC7648316

[ref-11] Harfield SG Davy C McArthur A : Characteristics of Indigenous primary health care service delivery models: a systematic scoping review. *Global Health.* 2018;14(1):12. 10.1186/s12992-018-0332-2 29368657 PMC5784701

[ref-12] Hong X Yan J Xu L : Relationship between nutritional status and frailty in hospitalized older patients. *Clin Interv Aging.* 2019;14:105–111. 10.2147/CIA.S189040 30666096 PMC6330965

[ref-13] Host A McMahon AT Walton K : Factors influencing food choice for independently living older people-a systematic literature review. *J Nutr Gerontol Geriatr.* 2016;35(2):67–94. 10.1080/21551197.2016.1168760 27153249

[ref-14] Kirk JW Nilsen P : Implementing evidence‐based practices in an emergency department: contradictions exposed when prioritising a flow culture. *J Clin Nurs.* 2016;25(3–4):555–565. 10.1111/jocn.13092 26818380 PMC4738684

[ref-15] Morley C Unwin M Peterson GM : Emergency department crowding: a systematic review of causes, consequences and solutions. *PLoS One.* 2018;13(8):e0203316. 10.1371/journal.pone.0203316 30161242 PMC6117060

[ref-16] O'Shaughnessy Í White S Smalle E : 106 Optimising Early Assessment and Intervention by Health and Social Care Professions in the ED: Preliminary Findings from the OPTIMEND RCT. *Age and Ageing.* 2019;48(Supplement_3):iii1–iii16. 10.1093/ageing/afz102.24

[ref-17] Ouzzani M Hammady H Fedorowicz Z : Rayyan-a web and mobile app for systematic reviews. *Syst Rev.* 2016;5(1):210. 10.1186/s13643-016-0384-4 27919275 PMC5139140

[ref-40] Peters MDJ Godfrey C McInerney P : Chapter 11: Scoping reviews.In: Aromataris E, Munn Z (Editors). *JBI Manual for Evidence Synthesis*, JBI,2020a. Reference Source

[ref-18] Peters MDJ Marnie C Tricco AC : Updated methodological guidance for the conduct of scoping reviews. *JBI Evid Synth.* 2020;18(10):2119–2126. 10.11124/JBIES-20-00167 33038124

[ref-19] Roe L Thomas S Trépel D : Trends in healthcare cover and healthcare use for older adults in Ireland during the austerity years of 2009-2016.Dublin: The Irish Longidudinal Study on Ageing.2018.

[ref-20] Shlisky J Bloom DE Beaudreault AR : Nutritional considerations for healthy aging and reduction in age-related chronic disease. *Adv Nutr.* 2017;8(1):17–26. 10.3945/an.116.013474 28096124 PMC5227979

[ref-21] Slawson DL Fitzgerald N Morgan KT : Position of the Academy of Nutrition and Dietetics: the role of nutrition in health promotion and chronic disease prevention. *J Acad Nutr Diet.* 2013;113(7):972–979. 10.1016/j.jand.2013.05.005 23790411

[ref-22] Stanga Z : Basics in clinical nutrition: Nutrition in the elderly. *e-SPEN, the European e-Journal of Clinical Nutrition and Metabolism.* 2009;6(4):e289–e299. 10.1016/j.eclnm.2009.06.019

[ref-23] Starr KNP McDonald SR Bales CW : Nutritional Vulnerability in Older Adults: A Continuum of Concerns. *Curr Nutr Rep.* 2015;4(2):176–184. 10.1007/s13668-015-0118-6 26042189 PMC4445877

[ref-24] Swan WI Pertel DG Hotson B : Nutrition care process (NCP) update part 2: developing and using the NCP terminology to demonstrate efficacy of nutrition care and related outcomes. *J Acad Nutr Diet.* 2019;119(5):840–855. 10.1016/j.jand.2018.10.025 30660633

[ref-25] Swan WI Vivanti A Hakel-Smith NA : Nutrition care process and model update: toward realizing people-centered care and outcomes management. *J Acad Nutr Diet.* 2017;117(12):2003–2014. 10.1016/j.jand.2017.07.015 28988837

[ref-26] Tittikpina NK Issa Ar Yerima M : Aging and nutrition: Theories, consequences, and impact of nutrients. * Curr Pharmacol Rep.* 2019;5(4):232–243. 10.1007/s40495-019-00185-6

[ref-27] Tricco AC Lillie E Zarin W : PRISMA extension for scoping reviews (PRISMA-ScR): checklist and explanation. *Ann Intern Med.* 2018;169(7):467–473. 10.7326/M18-0850 30178033

[ref-28] Tricco AC Lillie E Zarin W : A scoping review on the conduct and reporting of scoping reviews. *BMC Med Res Methodol.* 2016;16(1):15. 10.1186/s12874-016-0116-4 26857112 PMC4746911

[ref-29] Umegaki H Asai A Kanda S : Factors associated with unexpected admissions and mortality among low-functioning older patients receiving home medical care. *Geriatr Gerontol Int.* 2017;17(10):1623–1627. 10.1111/ggi.12943 28060439

[ref-30] van Tiel S Rood PP Bertoli-Avella AM : Systematic review of frequent users of emergency departments in non-US hospitals: state of the art. *Eur J Emerg Med.* 2015;22(5):306–315. 10.1097/MEJ.0000000000000242 25647038

[ref-31] Vivanti A Isenring E Baumann S : Emergency department malnutrition screening and support model improves outcomes in a pilot randomised controlled trial. *Emerg Med J.* 2015;32(3):180–183. 10.1136/emermed-2013-202965 24192128

