# Supplementary material for: What is the level of nutrition care provided to older adults attending emergency departments? A scoping review protocol
Source: HRB Open Res. 2022 Aug 31;5:7. Originally published 2022 Jan 18. [Version 2] doi: 10.12688/hrbopenres.13485.2 (PMC9459170; doi:10.12688/hrbopenres.13485.2)
Supplement: Peer review history

# Peer review: Reviewer response for version 2

**DOI:** 10.21956/hrbopenres.14871.r32753

**Published:** 2022-09-08

**Authors:** Adrienne M. Young

**Affiliations:** 1 Department of Nutrition and Dietetics, Royal Brisbane and Women's Hospital, Brisbane, QLD, Australia; 2 Centre for Health Services Research, University of Queensland, Brisbane, QLD, Australia

The authors have adequately addressed the previous feedback provided.

Is the study design appropriate for the research question?

Yes

Is the rationale for, and objectives of, the study clearly described?

Yes

Are sufficient details of the methods provided to allow replication by others?

Partly

Are the datasets clearly presented in a useable and accessible format?

Yes

Reviewer Expertise:

Malnutrition, hospital nutrition care, health services research

I confirm that I have read this submission and believe that I have an appropriate level of expertise to confirm that it is of an acceptable scientific standard.

# Peer review: Reviewer response for version 2

**DOI:** 10.21956/hrbopenres.14871.r32754

**Published:** 2022-09-01

**Authors:** Clare A. Corish

**Affiliations:** 1 School of Public Health, Physiotherapy and Sports Science, University College Dublin, Dublin, Ireland

No further comments.

Is the study design appropriate for the research question?

Yes

Is the rationale for, and objectives of, the study clearly described?

Yes

Are sufficient details of the methods provided to allow replication by others?

Yes

Are the datasets clearly presented in a useable and accessible format?

Not applicable

Reviewer Expertise:

I have a long-standing commitment to nutrition research activities, particularly in disease-related malnutrition/malnutrition in the older adult with research funding in this area from the Irish Health Service Executive, the Food Safety Promotion Board, safefood, the Department of Agriculture, Food and the Marine, and the Health Research Board. I have been workpackage co-lead on a large EU project investigating malnutrition in older adults (the Malnutrition in the Elderly Knowledge Hub). I am currently the national principal investigator on a large EU grant developing a dietary intervention to improve appetite in older adults at risk of malnutrition and have recently completed an Irish Health Research Board-funded project on the management of malnutrition in primary care. I was a member of the Irish Department of Health Guideline Development Group for the National Clinical Guideline 22, Nutrition screening and use of oral nutrition support for adults in the acute care setting in 2020, and the Food Safety Authority of Ireland Public Health Nutrition sub-committee that developed the Scientific Recommendations for Food-based Dietary Guidelines for Older Adults in Ireland in 2021.

I confirm that I have read this submission and believe that I have an appropriate level of expertise to confirm that it is of an acceptable scientific standard.

# Peer review: Reviewer response for version 1

**DOI:** 10.21956/hrbopenres.14703.r32398

**Published:** 2022-07-25

**Authors:** Clare A. Corish

**Affiliations:** 1 School of Public Health, Physiotherapy and Sports Science, University College Dublin, Dublin, Ireland

The proposed scoping review aims to identify the extent of nutrition care provided to older adults attending and subsequently discharged from emergency departments i.e., those not admitted directly from the emergency department into hospital. Nutrition is a modifiable factor for healthy ageing. Those who are malnourished have a lower quality of life, a greater need for healthcare, and are at the highest risk for frailty. Given the number of older adults who present, the emergency department provides an opportunistic setting to provide multidisciplinary malnutrition screening, assessment, and first-line interventions to manage malnutrition. This scoping review is timely, the methods used are clearly described and the results will be useful to inform healthcare practice both nationally and internationally. The review is broad, intended to capture grey as well as published literature, given the results of initial literature searches.

 I have one query relating to the inclusion criteria. In some places, an age of >65 years is specified and in other places, a mean age of >65 years. To my mind, these are quite different age categories. If the age is specified as >65 years, all in the population being investigated are aged 65 years and over. In contrast, a population with a mean age of >65 years could include people aged in their 50s if a proportion of the population is aged from 90-100+ years. It would be helpful if the authors could clarify precisely how they are categorising the study population and that consistent categorisation is used.

 I would also like to see dissemination/presentation of the research findings to the wider multidisciplinary team and not solely to dietitians, particularly if the anticipated follow on from the scoping review is to establish a process for malnutrition screening, assessment, and first-line intervention in the emergency department setting.

 In summary, this is a well-considered and written protocol for a valuable scoping review that is intended to inform nutrition care pathways into the integrated care of older adults. I look forward to its publication.

Is the study design appropriate for the research question?

Yes

Is the rationale for, and objectives of, the study clearly described?

Yes

Are sufficient details of the methods provided to allow replication by others?

Yes

Are the datasets clearly presented in a useable and accessible format?

Not applicable

Reviewer Expertise:

I have a long-standing commitment to nutrition research activities, particularly in disease-related malnutrition/malnutrition in the older adult with research funding in this area from the Irish Health Service Executive, the Food Safety Promotion Board, safefood, the Department of Agriculture, Food and the Marine, and the Health Research Board. I have been workpackage co-lead on a large EU project investigating malnutrition in older adults (the Malnutrition in the Elderly Knowledge Hub). I am currently the national principal investigator on a large EU grant developing a dietary intervention to improve appetite in older adults at risk of malnutrition and have recently completed an Irish Health Research Board-funded project on the management of malnutrition in primary care. I was a member of the Irish Department of Health Guideline Development Group for the National Clinical Guideline 22, Nutrition screening and use of oral nutrition support for adults in the acute care setting in 2020, and the Food Safety Authority of Ireland Public Health Nutrition sub-committee that developed the Scientific Recommendations for Food-based Dietary Guidelines for Older Adults in Ireland in 2021.

I confirm that I have read this submission and believe that I have an appropriate level of expertise to confirm that it is of an acceptable scientific standard.

# Response

**Published:** 2022-08-11

**Authors:** Anne Griffin

**Affiliations:** University of Limerick, Ireland

Thank you, Professor Corish for your time in reviewing our paper. We appreciate all of your comments and suggested changes.

 We have addressed each of the points you have raised and amended the paper accordingly as described below:

**Point 1: **Query relating to the inclusion criteria. In some places, an age of >65 years is specified and in other places, a mean age of >65 years.  It would be helpful if the authors could clarify precisely how they are categorising the study population and that consistent categorisation is used.

***Our Response*:** Thank you for your observation. We have amended the protocol to specify an age of > 65 years as our population of interest.

***Action*** **: **Pg. 6 & 7 - Two instances (section Eligibility Criteria describing “Population” and “Exclusion Criteria”) of text referring to “mean age” have been amended to clarify “age >65 years”.

**Point 2: **I would also like to see dissemination/presentation of the research findings to the wider multidisciplinary team and not solely to dietitians, particularly if the anticipated follow on from the scoping review is to establish a process for malnutrition screening, assessment, and first-line intervention in the emergency department setting.

***Our Response*** **: **Thank you for spotting this oversight on our behalf. We agree that the multidisciplinary team is essential to empower optimal nutrition care to older adults.

***Action*** **: **Pg. 10 - The text has been amended to state: “We will present our findings to healthcare professionals engaged in the service provision of  care for older adults in acute and frailty intervention teams to engage stakeholders to establish a process for malnutrition screening, assessment, and first-line intervention in the emergency department setting.”

 Kind regards,

 Anne Griffin,

 Corresponding author.

# Peer review: Reviewer response for version 1

**DOI:** 10.21956/hrbopenres.14703.r31843

**Published:** 2022-04-26

**Authors:** Adrienne M. Young

**Affiliations:** 1 Department of Nutrition and Dietetics, Royal Brisbane and Women's Hospital, Brisbane, QLD, Australia; 2 Centre for Health Services Research, University of Queensland, Brisbane, QLD, Australia

Thank you for this review which will be helpful for health professionals and researchers alike.

 The scoping review method is appropriate and well justified. The authors mention extracting the patient outcomes on p4 as a secondary finding, which is not typical for a scoping review and is not justified or described further. I would encourage the authors to consider if this fits within the scope of a scoping review, and if to be included this should be explained more fully and included in the data extraction form (i.e. Table 2). 

 The eligibility criteria are well described, however, the nutrition care at 72 hours is probably not relevant under the population as it is well covered under the concept. I would write the population as " *Older male and female adults (those with a mean age ≥65 years) who present to the ED and are subsequently discharged from emergency departments*." To this point, I wonder if " *admitted to hospital from ED*" should be included as part of the exclusion criteria. This is stated in the population, but I imagine that there are many studies that look at nutrition care across the continuum of care from ED to the hospital ward.

 The inclusion of grey literature is justified, however, a description of how Google searches will be managed should be included, e.g. how many pages/hits will be screened? Will you use any limiters (e.g. .gov.uk, etc).

 The protocol is well written. I noted only 2 typos/errors: " *MNA-SF*" is written as " *MNS-SF*"; and the second sentence in the introduction is a little hard to read (maybe better written as " *This can occur due to physiological factors (e.g. ...), psychosocial factors (e.g. ...), and personal resources (e.g. ...)*".

 All the best for completing your review.

Is the study design appropriate for the research question?

Yes

Is the rationale for, and objectives of, the study clearly described?

Yes

Are sufficient details of the methods provided to allow replication by others?

Partly

Are the datasets clearly presented in a useable and accessible format?

Yes

Reviewer Expertise:

Malnutrition, hospital nutrition care, health services research

I confirm that I have read this submission and believe that I have an appropriate level of expertise to confirm that it is of an acceptable scientific standard, however I have significant reservations, as outlined above.

# Response

**Published:** 2022-08-11

**Authors:** Anne Griffin

**Affiliations:** University of Limerick, Ireland

Thank you, Professor Young for your time in reviewing our paper.

 We appreciate all of your comments and suggested changes. We have addressed each of the points you have raised and amended the paper accordingly. This is now described below.

**Point 1:** The scoping review method is appropriate and well justified. The authors mention extracting the patient outcomes on p4 as a secondary finding, which is not typical for a scoping review and is not justified or described further. I would encourage the authors to consider if this fits within the scope of a scoping review, and if to be included this should be explained more fully and included in the data extraction form (i.e. Table 2). 

***Our Response*** **: **Thank you, we have considered this observation and agree that extracting patient outcomes is not required of a scoping review. Additionally, the stated objectives of the current study are to explore, map and describe nutrition care pathways initiated in the ED setting. Follow-up of patient outcomes is beyond the remit of these objectives and, indeed, is not carried out in reality from ED settings.

***Action: *** Pg. 6 - We have deleted the line: “We are not primarily interested in patient outcomes for this review but will chart these as a secondary finding.” From the section describing the development of inclusion criteria.

**Point 2**: The eligibility criteria are well described, however, the nutrition care at 72 hours is probably not relevant under the population as it is well covered under the concept. I would write the population as " *Older male and female adults (those with a mean age ≥65 years) who present to the ED and are subsequently discharged from emergency departments*."

***Our Response*:** Thank you, we have amended the sentence as you kindly suggested.

***Action: *** Pg. 6 - We have deleted the words “up to 72 hours post discharge to include early discharge planning phase” from the section titled ‘Population’. 

**Point 3: **I wonder if " *admitted to hospital from ED*" should be included as part of the exclusion criteria. This is stated in the population, but I imagine that there are many studies that look at nutrition care across the continuum of care from ED to the hospital ward.

***Our Response*: **Thank you, having subsequently screened a number of articles, we concur that nutrition screening is often not initiated until hospital ward admission.

***Action*: **Pg. 7 - “Articles in which nutrition care is initiated on a hospital ward and not in the ED.” Has been added to the exclusion criteria.

**Point 4**: The inclusion of grey literature is justified, however, a description of how Google searches will be managed should be included, e.g. how many pages/hits will be screened? Will you use any limiters (e.g. .gov.uk, etc).

***Our Response*:** Thank you for the comment. We found a lack of clear guidance on the management of Google searches in literature reviews. Based on the available information, our interpretation, taking a pragmatic approach to ensure the inclusion of health service policies and guidelines that are often not peer-reviewed we now describe our chosen method in more detail.

***Action*: **Pg. 8 - The following text with supporting references has been inserted to point 3 of the section ‘Search Strategy’: “As internet searching using Google.com displays results listed by relevance for the given search terms (Adams *et al.* 2016; Piasecki *et al.* 2018), the first 20 results yielded by the search string will be reviewed.  A custom range on the last ten years (2011 to 2021) will be the only limiter set.”

 References to add to list - Adams, J., Hillier-Brown, F.C., Moore, H.J. *et al.* Searching and synthesising ‘grey literature’ and ‘grey information’ in public health: critical reflections on three case studies. *Syst Rev* **5, **164 (2016). https://doi.org/10.1186/s13643-016-0337-y

 Piasecki J, Waligora M, Dranseika V. Google Search as an Additional Source in Systematic Reviews. Sci Eng Ethics. 2018 Apr;24(2):809-810. doi: 10.1007/s11948-017-0010-4. Epub 2017 Dec 16. PMID: 29249022; PMCID: PMC5876410.

**Point 5**: 2 typos/errors: " *MNA-SF*" is written as " *MNS-SF*"; and the second sentence in the introduction is a little hard to read (maybe better written as " *This can occur due to physiological factors (e.g. ...), psychosocial factors (e.g. ...), and personal resources (e.g. ...)*".

***Our Response*:** Thank you for spotting these typos and errors.

***Action*: **Pg. 5 - We have corrected MNS-SF to read MNA-SF.  We have amended the second sentence in the introduction as suggested.

 Pg. 4 -  It now reads “This can occur due to physiological factors (e.g. taste changes, poor dentition, loss of appetite, mobility and functional limitations), psychosocial factors (e.g. life course, food ideals and preferences, grief and bereavement), and personal resources (e.g. transport, disposable income, social supports) that influence food choice and intake (Stanga 2009; Host *et al.* 2016) among older adults. “

 Kind regards,

 Dr Anne Griffin,

 Corresponding author
